# Mass oscillations and matter wave’s phase and amplitude modulations of relativistic quantum particles induced by Heisenberg’s uncertainty principle

**DOI:** 10.1038/s41598-022-19344-9

**Published:** 2022-09-01

**Authors:** Jau Tang, Qiang Tang, Z. B. Hu

**Affiliations:** Institute of Technological Sciences, Wuhan University, Wuhan, 430072 Hubei China

**Keywords:** Matter waves and particle beams, Particle physics

## Abstract

We present a flip-flop dual-component model to treat quantum dynamics of relativistic particles with a rest mass and investigate the matter waves’ phase and amplitude modulations due to Heisenberg’s uncertainty principle. Their matter waves behave like a traveling Gaussian-shaped wave packet accompanied by a guiding pilot wave, and the phase modulations result in mass oscillations. These effects are more prominent for light-weighted elementary particles, such as neutrinos and electrons. This mechanism is solely due to the uncertainty principle and has nothing to do with the flavor-mixing of neutrinos. Simulations using neutrinos and electrons are presented, which indicate an oscillation period on the order of ps. This study primarily focuses on the predicted mass oscillations induced by the uncertainty principle. A slit-type interference experiment using neutrinos and electrons from reactors is proposed to test the predicted behaviors.

## Introduction

Heisenberg’s uncertainty principle is one of the essential concepts in quantum physics. Uncertainty between a pair of conjugate canonical variables, such as position and momentum, time and energy, etc., plays a critical role in governing the sub-atomic world's quantum behaviors, which are so different from the classical understanding of the macroscopic world in daily life. Neutrinos are the most mysterious and elusive particles among all known elementary particles^1^. Even though one hundred trillion of these particles pass through the human body per second, humans have scarce information about them. It is known that neutrinos move close to light speed and have a negligibly small rest mass. Because these particles only involve weak interactions, their detection is difficult. However, their existence and physical properties are closely tied to understanding the origin of our universe and its evolution. Therefore, studies of neutrinos have remained a hot and essential subject in physics. During the past decades, observations of neutrino mass oscillations have generated tremendous research interest. Recognizing such efforts led to the 2002 Nobel Prize for key researchers, Kajita and McDonald^2,3^. Besides their essential contributions, extensive research has been conducted worldwide on theoretical and experimental fronts^4–8^, such as the Super-Kamiokande Observatory and the Sudbury Neutrino Observatory^4,9^, and more recently MINOS and DUNE facilities^10,11^.

## Theory

### Matter waves of relativistic particles in 2D space–time

In this report we only consider matter-wave propagation of a relativistic particle and do not need to consider spin-dependent interactions. Even though neutrinos and electrons are spin-1/2 particles, and they follow descriptions of Dirac’s equation, like all other leptons, we present here a simple model to describe relativistic scalar particles, borrowing the approach from Dirac by linearizing the wave equation in space and time^12^, with neither a spin nor an anti-particle component. We consider a flip-flop dual-component model involving a set of linearly coupled first-order differential equations in space–time of two real-valued functions, as given in the following

1 $$ \begin{aligned} \frac{\partial }{\partial t}f\left( {x,t} \right) & = \omega g\left( {x,t} \right) \\ \frac{\partial }{\partial t}g\left( {x,t} \right) & = - \omega f\left( {x,t} \right) \\ \frac{\partial }{\partial x}f\left( {x,t} \right) & = - kg\left( {x,t} \right) \\ \frac{\partial }{\partial x}g\left( {x,t} \right) & = kf\left( {x,t} \right). \\ \end{aligned} $$

The above equation describes the flip-flop exchange between two components $g(x,\,t)\,$ in time and space. The solution to the above equation represents a plane wave with an amplitude and a phase $\phi$. This wave function can describe general wave propagation, such as water, sound, and electromagnetic waves. It can also describe de Broglie’s matter waves if one replaces $\omega$ and uses the de Broglie–Einstein relation^12^ of

2 $$ \begin{aligned} \omega & = c{{\sqrt {\hbar^{2} k^{2} + m_{0}^{2} c^{2} } } \mathord{\left/ {\vphantom {{\sqrt {\hbar^{2} k^{2} + m_{0}^{2} c^{2} } } \hbar }} \right. \kern-\nulldelimiterspace} \hbar } \\ k & = p/\hbar . \\ \end{aligned} $$

The physical interpretation of these real-valued functions, i.e., $f\left( {x,t} \right)$ and $g\left( {x,t} \right)$, represents the field strength in the quantum field theory concept. Such a set of coupled rate equations could lead to the Klein–Gordon equation for a relativistic particle and Schroedinger equation for a non-relativistic particle. Our simple flip-flop dual-component model offers an approach to derive either equation for the wave function for relativistic and non-relativistic spinless particles. More details are given in the Supplementary Materials.

According to Heisenberg’s uncertainty principle, there is a lack of a priori knowledge about a particle’s initial momentum in the natural microscopic world. Thus, we consider a Gaussian probability distribution with a center and a variance $\Delta k$. Using the Taylor expansion of $\omega \left( k \right)$ with $k$ around $k_{0}$, one obtains the normalized relativistic wave function as given by

3 $$ \begin{aligned} \Psi \left( {x,t} \right) & = \frac{1}{{\sqrt {2\pi } \Delta k}}\int\limits_{ - \infty }^{\infty } {dk} \,\exp \left( { - {{\left( {k - k_{0} } \right)^{2} } \mathord{\left/ {\vphantom {{\left( {k - k_{0} } \right)^{2} } {2\Delta k^{2} }}} \right. \kern-\nulldelimiterspace} {2\Delta k^{2} }} + i\,kx - i\,t\omega \left( k \right) + i\phi } \right) \\ & = \frac{1}{{\sqrt {2\pi } \,\Delta k}}\exp \left( {ik_{0} x - i\,t\omega \left( {k_{0} } \right)} \right)\int\limits_{ - \infty }^{\infty } {dK} \,\exp \left( { - {{K^{2} } \mathord{\left/ {\vphantom {{K^{2} } {2\Delta k^{2} }}} \right. \kern-\nulldelimiterspace} {2\Delta k^{2} }} + i\,Kx - i\,t\left( {\omega \left( {K + k_{0} } \right) - \omega \left( {k_{0} } \right)} \right)} \right). \\ \end{aligned} $$

where $K\, = \,k\, - \,k_{0}$ and the constant phase $\phi$ is omitted. Using Taylor’s approximation of $\omega \left( {K + k_{0} } \right)$ for a small $K$ one can further simplify the above equation according to the steepest descent method

4A $$ \begin{aligned} \Psi \left( {x,t} \right) & = \frac{{\exp \left( {ik_{0} x - i\,t\,m\,c^{2} /\hbar } \right)}}{{\sqrt {2\pi } \,\Delta k}} \\ & \quad \times \int\limits_{ - \infty }^{\infty } {dK} \,\exp \left( { - {{K^{2} } \mathord{\left/ {\vphantom {{K^{2} } {2\Delta k^{2} }}} \right. \kern-\nulldelimiterspace} {2\Delta k^{2} }} + iKx - ict\frac{{K^{2} + 2Kk_{0} }}{{\sqrt {K^{2} + 2Kk_{0} + m^{2} c^{2} /\hbar^{2} } + mc/\hbar }}} \right) \\ & \approx \exp \left( {i\,k_{0} x - i\omega_{0} t} \right)\frac{1}{{\sqrt {1 + i\,t\Delta k^{2} \hbar /m} }}\exp \left( { - \frac{{\left( {x - v_{0} t} \right)^{2} \Delta k^{2} }}{{1 + i\,t\Delta k^{2} \hbar /m}}} \right) \\ & = \frac{{\exp \left( {i\,k_{0} x - i\,t\,m\,c^{2} /\hbar } \right)}}{{\sigma^{2} \left( t \right)}}\exp \left( { - \frac{{\left( {x - v_{0} t} \right)^{2} \Delta k^{2} }}{{\sigma^{2} \left( t \right)}}e^{ - i\xi \left( t \right)} - i\xi \left( t \right)} \right) \\ \end{aligned} $$

where $$v_{0} = \hbar k_{0} /m,\quad \omega_{0} = mc^{2} /\hbar$$ and

4B $$ \begin{aligned} \sigma^{2} \left( t \right) & = 1 + \left( {{{t\Delta k^{2} \hbar } \mathord{\left/ {\vphantom {{t\Delta k^{2} \hbar } m}} \right. \kern-\nulldelimiterspace} m}} \right)^{2} \\ \xi \left( t \right) & \equiv \frac{1}{2}\tan^{ - 1} \left( {{{t\Delta k^{2} \hbar } \mathord{\left/ {\vphantom {{t\Delta k^{2} \hbar } m}} \right. \kern-\nulldelimiterspace} m}} \right). \\ \end{aligned} $$

The second moment indicates in time, and the additional time-dependent phase $\xi \left( t \right)$ will be shown later to induce mass oscillations. The solution of the wave function in Eq. (4) represents a Gaussian-shaped wave packet traveling accompanied by a guiding pilot wave as given by $\exp \left( {i\,k_{0}^{{}} x - \,i\,\omega_{0} t} \right)\,$. This allows one to conceptually visualize the particle as a traveling wave packet with amplitude modulation and travels with a pilot wave at the same speed. This picture is analogous to the concept of amplitude modulation (AM) radio signal riding on an electromagnetic carrier wave. However, for a relativistic particle with a rest mass, the wave function solution can be visualized as a traveling wave packet with amplitude and frequency modulation accompanied by a pilot wave at the same speed $v_{0}$. These two terms of amplitude and phase modulations are insignificant if the particles have a large rest mass and a small $\Delta k^{2}$, i.e., a low uncertainty in the wave vector distribution or a large uncertainty in the particle’s location. However, the modulations in both amplitude and phase can be significant for light-weighted particles, such as neutrinos or beta-decay electrons, but with a large due to their origins within nuclei.

The approach presented here is superior to the pilot wave theory of de Broglie and Bohm^13,14^, as their theory cannot be extended to relativistic particles. Our flip-flop dual model involves the first-order derivative in 1D space and time as Dirac's theory and can be used to treat relativistic scalar particles. In addition, our trajectory approach is also better than the wave equation theory of Schrödinger, which can only treat non-relativistic particles. It can be shown that from Eqs. (1) and (2) one can easily re-derive the Klein–Gordon equation for a relativistic scalar particle and the Schrödinger equation for particles at a low speed. Therefore, our simple flip-flop model involving a set of linearly coupled first-order differential equations in space–time, in conjunction with the de Broglie-Einstein relation, provides more clear physical pictures with a simpler trajectory approach than that of the de Broglie–Bohm pilot-wave theory^13,14^. In a recent work, we have applied the flip-flop dual-component model to treat Young’s double-slit interference involving single photons^15^. We have elucidated the actual mechanism and disproved the necessity of the widely-accepted self-interference hypothesis. We showed that self-interference is an illusion caused by long coherence length of single photons and their highly delocalized wave packets. The primary purpose of proposing the flip-flop dual-component model in 2D space–time is to treat mass oscillations due to Heisenberg’s uncertainty principle for a relativistic neutrino or electron. This dual-component model with linearized time and space derivatives could be extended to 4D space–time to treat other elementary particles, such as leptons, quarks, and gauge bosons. Further development and new results will be published elsewhere in the future.

### Interference of two beams

Now we consider a slit-type experiment involving neutrinos in MINOS or DIME facilities^10,11^. In the investigation, a neutrino beam was sent from the Fermi Lab to a detector in Northern Minnesota. The neutrinos sometimes disappeared in flight and confirmed the observation arose from the wave phenomena, similar to Young’s double-slit interference of photons. Let us use Eq. (4A) to analyze the interference effects of two neutrino beams whose wave functions are represented by $\Psi_{1} (t)\,$ and $\Psi_{2} (t)\,$, respectively. The interference intensity of two beams reflects the intensity from the summation of two wave functions with a difference between two neutrino beams with a different emission time delay $T$ and an average arrival time $\tau$ for the beams to reach a distant detector. According to the well-known formula for the double-slit intensity interference and with $t\,,\,\,\tau \,\,\, > > \,\,T$ the wave equation for two beams can be approximated by

5 $$ \begin{aligned} \Psi_{1} \left( {x,t} \right) & = \frac{{\exp \left( {i\left( {v_{0}^{2} \tau - c^{2} \left( {t - T/2} \right)} \right)m/\hbar } \right)}}{{\sqrt {1 + i\,t\Delta k^{2} \hbar /m} }}\exp \left( { - \frac{{\left( {\tau - \left( {t - T/2} \right)} \right)^{2} v_{0}^{2} \Delta k^{2} }}{{1 + i\,t\,\Delta k^{2} \hbar /m}}} \right) \\ \Psi_{2} \left( {x,t} \right) & = \frac{{\exp \left( {i\left( {v_{0}^{2} \tau - c^{2} \left( {t + T/2} \right)} \right)m/\hbar } \right)}}{{\sqrt {1 + i\,t\Delta k^{2} \hbar /m} }}\exp \left( { - \frac{{\left( {\tau - \left( {t + T/2} \right)} \right)^{2} v_{0}^{2} \Delta k^{2} }}{{1 + i\,t\,\Delta k^{2} \hbar /m}}} \right) \\ \end{aligned} $$

The overall intensity from these two beams is given by

6A $$ I\left( {\,t,\,T,\,\tau } \right) = \left| {\Psi_{1} \left( {x,\,t} \right) + \Psi_{2} \left( {x,\,t} \right)} \right|^{2} = I_{1} \left( {t,\,T,\,\tau } \right) + I_{2} \left( {t,\,T,\,\tau } \right) + I_{{\text{int}}} \left( {t,\,T,\,\tau } \right) $$

where

6B $$ \begin{gathered} I_{1} \left( {t,\,T,\,\tau } \right) = \frac{1}{{1 + \left( {t\,\Delta k^{2} \hbar /m} \right)^{2} }}\,\exp \,\left( { - \frac{{2\left( {\tau - t + T/2} \right)^{2} v_{0}^{2} \Delta k^{2} }}{{1 + \left( {t\,\Delta k^{2} \hbar /m} \right)^{2} }}} \right) \hfill \\ I_{2} \left( {t,\,T,\,\tau } \right) = \frac{1}{{1 + \left( {t\,\Delta k^{2} \hbar /m} \right)^{2} }}\,\exp \,\left( { - \frac{{2\left( {\tau - t - T/2} \right)^{2} v_{0}^{2} \Delta k^{2} }}{{1 + \left( {t\,\Delta k^{2} \hbar /m} \right)^{2} }}} \right) \hfill \\ \end{gathered} $$

and the interference term

6C $$ \begin{aligned} & I_{{\text{int}}} \left( {t,T,\tau } \right) = \frac{2}{{1 + \left( {t\,\Delta k^{2} \hbar /m} \right)^{2} }}\,\exp \left( { - \frac{{2\left( {\left( {\tau - t} \right)^{2} + T^{2} /4} \right)v_{0}^{2} \Delta k^{2} }}{{1 + \left( {t\,\Delta k^{2} \hbar /m} \right)^{2} }}} \right)\,\cos \,\left( {\Phi \left( {T,t - \tau ,\Delta k^{2} } \right)} \right) \\ & \Phi \left( {T,t - \tau ,\Delta k^{2} } \right) \equiv 2T\left( {t\, - \,\tau } \right)\left( {\frac{{\left( {t\,\Delta k^{2} \hbar /m} \right)v_{0}^{2} \Delta k^{2} }}{{1 + \left( {t\,\Delta k^{2} \hbar /m} \right)^{2} }}} \right) \\ \end{aligned} $$

From the above equation, one can see that the oscillation angular frequency $\Omega$ along the time axis of $t\, - \,\tau$ is given approximately by $\Omega = 2T\,\left( {{{\left( {\tau \,\Delta k^{2} \hbar /m} \right)v_{0}^{2} \Delta k^{2} } \mathord{\left/ {\vphantom {{\left( {\tau \,\Delta k^{2} \hbar /m} \right)v_{0}^{2} \Delta k^{2} } {\left( {1 + \left( {\tau \,\Delta k^{2} \hbar /m} \right)^{2} } \right)}}} \right. \kern-\nulldelimiterspace} {\left( {1 + \left( {\tau \,\Delta k^{2} \hbar /m} \right)^{2} } \right)}}} \right).$ This formula indicates that the mass oscillations will be insignificant for particles with a huge mass or a minimal uncertainty in $\Delta k^{2}$. Therefore, light-weighted neutrinos and electrons are the best candidates for such uncertainty-induced mass oscillation experiments. A high-speed oscillating phase term represents the carrier pilot-wave due to mass-energy being omitted in the derivation. We are primarily concerned about the oscillations due to small mass changes induced by the Heisenberg uncertainty principle. The above equation for mass oscillations is the main result of this work. The following section will present simulation results based on Eq. (6) for neutrinos and electrons.

## Results and discussion

**Figure 1:**
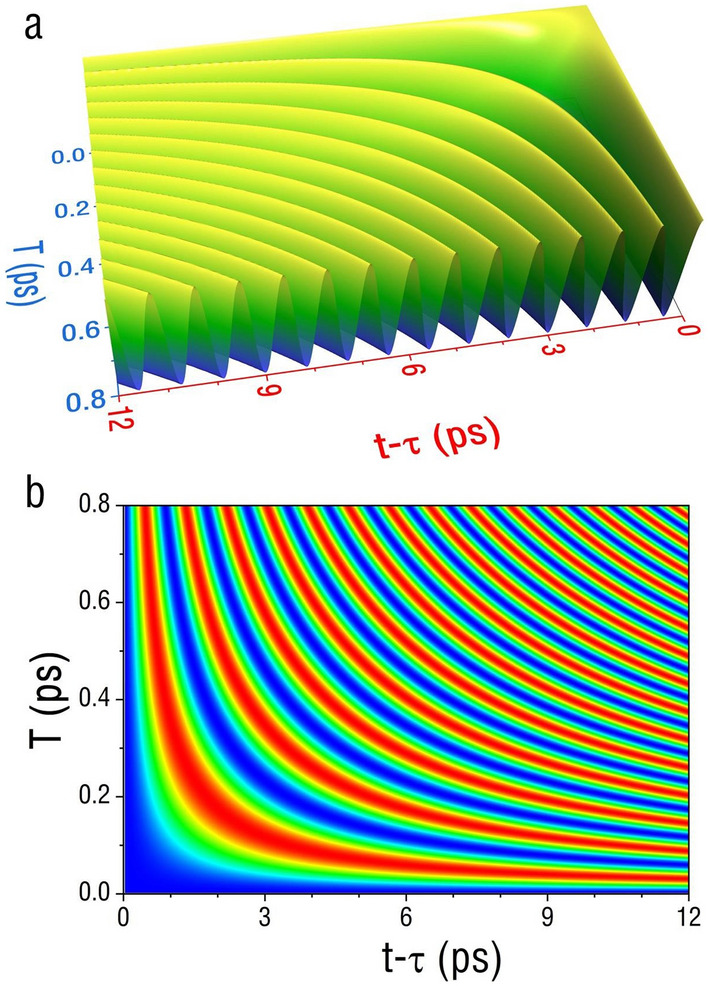
The mass oscillation behaviors of electron-neutrinos. (**a**) A 3D plot of the dependence of $\cos \,\left( {\Phi \left( {T,\,t\, - \,\tau ,\,\,\Delta k^{2} } \right)} \right)$ for the neutrino case, on $T$, the time interval between two neutrino beams, and $t\, - \,\tau \,$, the average time for two beams to reach a detector. (**b**) A contour plot of the same function. Both figures illustrate the intensity oscillations from two interfering neutrino beams, reflecting the mass oscillations induced by the uncertainty in neutrino’s initial wave vector $\Delta k$. In this simulation, we consider electron-neutrino from reactors with relativistic mass-energy of 0.5 MeV, $\Delta k$ of 10^10^ m^−1^ and $\tau$ of 2.5 ms. The oscillation period appears to be on the order of sub-ps along the time axis of $t\, - \,\tau \,$.

Here we discuss the mass oscillation results based on Eq. (6C) for both cases of neutrinos and electrons. Figure 1a,b are 3D perspective and cross-section line curve plots of the same function, showing mass oscillations of neutrinos.

**Figure 2:**
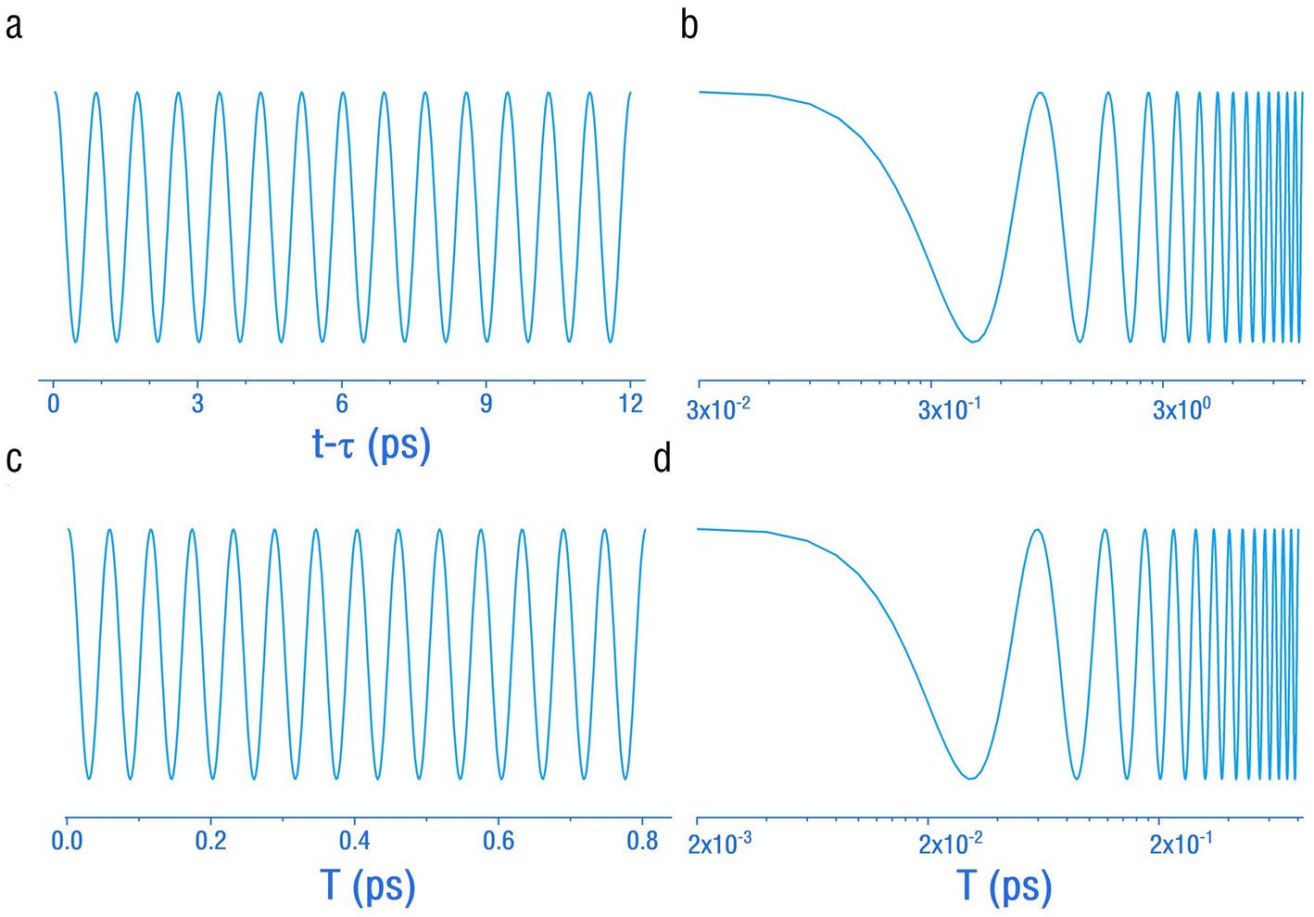
The oscillation behaviors of the interference intensity for electron-neutrinos. (**a**) A cross-section line curve plot of $\cos \,\left( {\Phi \left( {T,\,t\, - \,\tau ,\,\,\Delta k^{2} } \right)} \right)$ versus $t\, - \,\tau \,$ in the ordinary time scale. (**b**) A similar plot but in a logarithmic time unit. (**c**) A cross-section line curve plot of $\cos \,\left( {\Phi \left( {T,\,t\, - \,\tau ,\,\,\Delta k^{2} } \right)} \right)$ versus $T\,$ in the ordinary time scale. (**d**) A similar plot but in a logarithmic time unit. Oscillations are present in both time directions, reflecting the mass oscillations induced by Heisenberg’s uncertainty principle. The oscillation period along $t\, - \,\tau \,$ is found to be on the order of ps.

In the following Fig. 2a, a 3D plot of the dependence of $\cos \,\left( {\Phi \left( {T,\,t\, - \,\tau ,\,\,\Delta k^{2} } \right)} \right)$ on the time interval $T$ and $\Delta k^{2}$ is illustrated. Subplot (b) is a contour plot of the same data set. Subplots (c) and (d) are 3D and contour plots as a function $t\, - \,\tau \,$. Mass oscillations occur along both axes.

For typical electron neutrinos from reactors with relativistic mass-energy about 0.5 MeV, $\Delta k$ of 2.5 × 10^11^ m^−1^, the oscillation period appears to be on the order of ps. ln Fig. 1, we used 0.5 MeV in the simulations to reflect an increase of its relativistic mass-energy by at least one million times than its rest mass-energy. We also used $\tau$ of 2.5 ms, assuming an approximate time needed for the neutrinos to travel to the MINOS facility from the Fermi Lab. These values are used just for demonstration to help visualize the oscillation behavior due to the Heisenberg uncertainty principle. A more significant uncertainty can cause a much higher mass oscillation frequency for neutrinos produced in the atmosphere due to high-energy cosmic rays.

**Figure 3:**
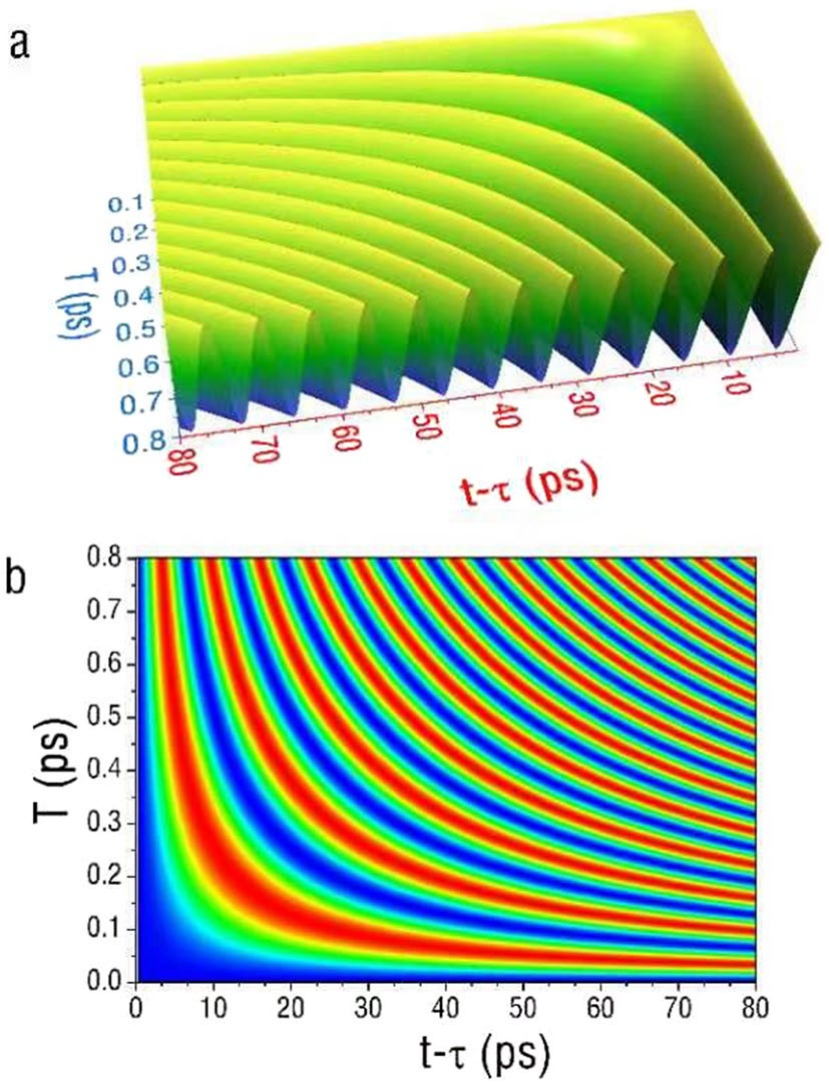
The mass oscillation behaviors of electrons. (**a**) A 3D plot of the dependence of $\cos \,\left( {\Phi \left( {T,\,t\, - \,\tau ,\,\,\Delta k^{2} } \right)} \right)$ for the electron case, on $T$, and $t\, - \,\tau \,$. (**b**) A contour plot of the same function. Both figures illustrate the intensity oscillations from two interfering electron beams, reflecting the mass oscillations induced by the uncertainty in the electron’s initial wave vector $\Delta k$. In this simulation, we consider neutrino from reactors with relativistic mass-energy of 0.55 MeV, $v_{0}$ = 1.1 × 10^8^ m/s, $\Delta k$ of 10^10^ m^−1^and $\tau$ of 0.91 µs for traveling 1 km to a detector. The oscillation period appears to be on the order of ps along the time axis of $t\, - \,\tau \,$.

Besides the electron neutrinos and muon neutrinos we illustrated previously, we also investigate the electron case to analyze the effects of the Heisenberg uncertainty principle on electron mass oscillations. For the electron case, the dependence of the interference intensity on $T\,$ and $\Delta k$ is illustrated in Fig. 3.

In this example, we consider a high-energy electron with relativistic mass-energy of about 0.55 MeV, compared to its rest mass energy of 0.51 MeV, and $\Delta k$ of 10^10^ m^−1^. The oscillation period along $t\, - \,\tau \,$ is found to be on the order of 10 ps. Due to the difference in their kinetic parameters, even though electrons exhibit similar behaviors as neutrinos, the oscillation periods along both time axes differ in magnitude.

## Conclusions

In conclusion, this work examines the matter wave’s phase and amplitude modulations of relativistic particles due to Heisenberg’s uncertainty principle. According to our analysis, all quantum particles with a rest mass possess these dynamics behaviors to some extent but are more pronounced for very light-weighted elementary particles such as neutrinos and electrons. To illustrate such dynamic quantum phenomena of the matter waves of relativistic particles more clearly, we conduct extensive simulations. Using both neutrinos and electrons as examples we demonstrate their mass oscillation behaviors due to this mechanism. Our theoretical analysis presents a simple flip-flop dual-component model involving a set of coupled first-order differential equations in 2D space–time with equal footing for both space and time. As shown in Eq. (5), the solution to this flip-flop dual-component model represents a Gaussian-shaped wave packet accompanied by a guiding pilot wave, analogous to an AM/FM radio signal riding on a carrier electromagnetic wave. Our simple relativistic quantum-field theory approach provides a more precise physical picture than the pilot wave theory of de Broglie and Bohm^13,14^, which has met difficulties in extending beyond the non-relativistic regime. According to our analysis, an initial uncertainty in a particle’s momentum leads to amplitude and phase modulations. Such modulation effects are more prominent for light-weighted particles such as neutrinos and electrons. These predicted effects could be tested in future slit-type interference experiments using neutrinos or electrons from reactors. Typical neutrinos generated in reactors have an energy of about 0.5 MeV, although their rest mass is estimated to be smaller than 0.8 eV. According to the Heisenberg uncertainty principle, such uncertainty in Eq. (4) this mechanism can significantly affect the amplitude and phase of the wave packet. In Eq. (6), we derive this work’s most crucial result, which predicts the mass oscillation behavior solely induced by the Heisenberg uncertainty principle. Such oscillation is most pronounced for light-weighted elementary particles, such as neutrinos and electrons. This dynamic behavior is predicted to be present in all quantum particles with a rest mass. But it is more pronounced for very light-weighted relativistic particles that possess a rather large uncertainty in the particle’s initial momentum. Therefore, neutrinos and electrons are excellent candidates for experimental tests for the predictions made in this work. Hopefully, such effects could be tested by the slit-type interference experiment using neutrinos as in the MINOS^10^ facility or using electrons from reactors.

## Method

The datasets used in producing three figures were obtained from MATLAB algorithms we wrote using Eq. (6) for calculations. The datasets generated and analyzed during the current study are not publicly available because all simulated data curves could be obtained by anyone quite straightforwardly.

## Supplementary Information

Supplementary Information.

## Data Availability

The Origin files for each figure and the data are available from the corresponding author upon reasonable request. The program listings in MATLAB are also available upon request.
