# Supplementary material for: Mass oscillations and matter wave’s phase and amplitude modulations of relativistic quantum particles induced by Heisenberg’s uncertainty principle
Source: Sci Rep. 2022 Sep 1;12:14881. doi: 10.1038/s41598-022-19344-9 (PMC9437067; doi:10.1038/s41598-022-19344-9)
Supplement: Supplementary file 1 — Supplementary Information. [file 41598_2022_19344_MOESM1_ESM.pdf]

Supplementary Materials

This Supplementary Material details the flip-flop dual-component model and its relations to the Klein-Gordon and **Schrödinger equation.** First, we offer a simple dual-component model to describe quantum behavior of relativistic particles in 2D space-time. Let us consider a pair of real functions to describe this binary system with dual components. The most elementary equation is a set of linearly coupled first-order differential equations in time and space as given by

$$\begin{aligned} &\frac{\partial}{\partial t} f(x,t) = \omega g(x,t) \\ &\frac{\partial}{\partial t} g(x,t) = -\omega f(x,t) \\ &\frac{\partial}{\partial x} f(x,t) = -k\, g(x,t) \\ &\frac{\partial}{\partial x} g(x,t) = k\, f(x,t) \end{aligned} \qquad \text{(S1)}$$

The above equation describes flip-flop dynamics in time and space between two components $f(x,t)$ and $g(x,t)$. The above rate equation in 2D space-time has equal footing in space and time, analogous to Dirac's treatment of a relativistic electron, except that he considered 4D space-time and introduced gamma matrices or spinor matrices that lead to the electron spin. With two fundamental parameters frequency ω. And wave vector k. These coupled equations describe oscillations in time and space with a solution and, represent a wave traveling along the x-axis, where A is an amplitude and ϕ a phase. By changing either or the solution represents a wave traveling in an opposite direction. A photon's most fundamental physical properties are frequency and light velocity c $\omega = kc$. For an acoustic wave, the velocity c is replaced by sound velocity. For a relativistic particle with a rest mass; the most fundamental physical parameters are its rest mass and momentum. Based on de Broglie's hypothesis of matter-wave duality and Einstein's relativity theory of mass-energy equivalence

$$\begin{aligned} &\omega = c\sqrt{k^2 + m_0^2 c^2 / \hbar^2} \\ &k = p / \hbar \end{aligned} \qquad \text{(S2)}$$

one could replace the frequency and wave vector in eq. (1) by the above relations. For a photon without a rest mass, the solution of Eq. (S1) becomes

$$\begin{aligned} &f(x,t) = A\cos(k(x - ct) + \phi) \\ &g(x,t) = A\sin(k(x - ct) + \phi) \end{aligned} \qquad \text{(S3)}$$

By defining a complex function then, one has $\Psi(x,t) = e^{i(k(x-ct)+\phi)}$ which represents the complex electric or magnetic field solution for a light wave. For a relativistic particle, one can derive from Eq. (S1)

$$\frac{\partial^2}{\partial t^2}\Psi(x,t) = -\frac{m_0^2 c^4}{\hbar^2}\Psi(x,t) + c^2\frac{\partial^2}{\partial x^2}\Psi(x,t) \tag{S4}$$

Using the dual-component model, we could re-derive the above Klein-Gordon equation for a scalar particle with a rest mass m_0. Unlike this K-G equation which involves 2nd derivatives in space and time of a complex-value wave function, our dual-component model in Eq. (S1) involves only the 1st order derivative with a real-value function.

For a free non-relativistic particle one has $\omega \approx m_0 c^2/\hbar + \hbar k^2/2m_0$, one can reduce eq. (1) to

$$\begin{aligned}
\frac{\partial}{\partial t} f(x,t) &= \left(\frac{m_0 c^2}{\hbar} + \frac{\hbar k^2}{2m_0}\right) g(x,t) \\
\frac{\partial}{\partial t} g(x,t) &= -\left(\frac{m_0 c^2}{\hbar} + \frac{\hbar k^2}{2m_0}\right) f(x,t) \\
\frac{\partial}{\partial x} f(x,t) &= -k\, g(x,t) \\
\frac{\partial}{\partial x} g(x,t) &= k\, f(x,t)
\end{aligned} \tag{S5}$$

The fast oscillating dependence in the above rate equation can be transformed away by redefining a new set of functions $F(x,t) \equiv \cos(m_0 c^2 t/\hbar) f(x,t) + \sin(m_0 c^2 t/\hbar) g(x,t)$ and $G(x,t) \equiv -\sin(m_0 c^2 t/\hbar) f(x,t) + \cos(m_0 c^2 t/\hbar) g(x,t)$, one has

$$\begin{aligned}
\frac{\partial}{\partial t} F(x,t) &= \frac{\hbar k^2}{2m_0} G(x,t) \\
\frac{\partial}{\partial t} G(x,t) &= -\frac{\hbar k^2}{2m_0} F(x,t) \\
\frac{\partial}{\partial x} F(x,t) &= -k\, G(x,t) \\
\frac{\partial}{\partial x} G(x,t) &= k\, F(x,t)
\end{aligned} \tag{S6}$$

with $\Psi(x,t) \equiv F(x,t) + i\,G(x,t)$ one can obtain

$$i\hbar\frac{\partial}{\partial t}\Psi(x,t) = -\frac{\hbar^2}{2m_0}\frac{\partial^2}{\partial x^2}\Psi(x,t) \tag{S7}$$

Here we pedagogically re-derive the **Schrödinger** equation. We use a simple two-component model involving elementary linearly coupled differential equations and Planck-Einstein-de Broglie relations.

The primary purpose of proposing the flip-flop dual-component model in 2D space-time is to treat mass oscillations due to Heisenberg's uncertainty principle for a relativistic neutrino or electron. This dual-component model with linearized time and space derivatives could be extended to 4D space-time to treat other elementary particles, such as leptons, quarks, and gauge bosons. Further development and new results will be published elsewhere in the future.
